# Clinical and Proteomic Associations of SARS-CoV-2 Infection and COVID-19 Vaccination in Multimorbid Patients: A Cross-Sectional Observational Study

**DOI:** 10.3390/ijms26168007

**Published:** 2025-08-19

**Authors:** Anett Hudák, Aladár Pettko-Szandtner, Annamária Letoha, Tamás Letoha

**Affiliations:** 1 Pharmacoidea Ltd., H-6726 Szeged, Hungary; anett.hudak@pharmacoidea.eu; 2 Biological Research Centre of the Hungarian Academy of Sciences, H-6726 Szeged, Hungary; pettko-szandtner.aladar@brc.hu; 3 Albert Szent-Györgyi Clinical Center, Department of Medicine, Faculty of Medicine, University of Szeged, H-6720 Szeged, Hungary; letoha.annamaria@med.u-szeged.hu

**Keywords:** COVID-19, vaccination, comorbidities, clinical outcomes, proteomics, mass spectrometry, vaccine safety and efficacy, biomarkers

## Abstract

Vaccines played a crucial role in the COVID-19 pandemic, but their long-term biological effects and efficacy in vulnerable populations remain under intensive investigation. This study assessed clinical outcomes, comorbidities, and systemic biomarker and proteomic profiles in 366 multimorbid patients, stratified into four groups based on SARS-CoV-2 infection and vaccination status (COV+ vac+, COV+ vac−, COV− vac+, COV− vac−). Clinical and laboratory data, including comorbidities and relevant biomarkers, were collected. Proteomic analysis using mass spectrometry was performed to identify molecular changes associated with infection and vaccination. Statistical analyses examined associations between clinical status, biomarkers, and patient outcomes. As most participants received mRNA-based vaccines, the results primarily reflect responses to spike protein-expressing platforms. Biomarkers of cardiac and renal stress—namely proBNP and carbamide—were elevated in vaccinated individuals. Five deaths occurred in the COV+ vac+ group and two in the COV+ vac− group, most of which were attributed to exacerbations of pre-existing chronic diseases rather than to COVID-19 pneumonia. Protection against breakthrough infections waned over time, particularly beyond 200 days post-vaccination. Mass spectrometry identified proteins such as actin, fibrinogen chains, and SAA2 as potential diagnostic targets. Although the cross-sectional observational design limits the ability to draw causal inferences, the observed waning immunity and potential systemic alterations in vaccinated multimorbid patients highlight the importance of longitudinal follow-up to guide tailored immunization strategies and post-vaccination monitoring in high-risk groups.

## 1. Introduction

Severe acute respiratory syndrome coronavirus 2 (SARS-CoV-2), the virus responsible for COVID-19, emerged in late 2019 and rapidly escalated into a global health crisis, affecting millions worldwide [1,2]. Beyond the acute respiratory symptoms, the pandemic has caused long-term health, economic, and social challenges, highlighting its multifaceted impact on societies globally [3]. COVID-19 primarily manifests in respiratory symptoms, including cough, difficulty breathing, and pneumonia. However, systemic complications, such as myocarditis, arrhythmias, thrombosis, and multi-organ damage, are of particular concern in severe cases [4,5]. Hyperactivation of the immune system in these cases often leads to excessive inflammatory responses, or cytokine storms, which are implicated in severe disease progression and fatal outcomes [6]. Furthermore, the emergence of SARS-CoV-2 variants with differing levels of transmissibility and virulence has further complicated public health responses and mitigation efforts [7].

In response to these ongoing challenges, global research efforts have focused on developing effective vaccines, antiviral therapies, and advanced diagnostics [8]. Despite the scale of widespread vaccination campaigns, a critical gap remains in understanding the broader biological effects of COVID-19 vaccines, particularly in individuals with pre-existing conditions [9]. Comorbidities such as cardiovascular disease (CVD), obesity, and hypertension (HT) are known risk factors for severe COVID-19 outcomes, yet their interaction with vaccination and SARS-CoV-2 infection requires further investigation [10]. Unraveling these intricate relationships demands interdisciplinary approaches, integrating molecular, biochemical, and clinical data [11]. Individual variability, including genetic and biochemical factors, adds complexity to understanding these relationships, necessitating molecular-level analyses to gain a deeper insight into disease mechanisms.

Mass spectrometry has emerged as a key tool in unraveling the molecular underpinnings of infectious diseases, including COVID-19. Mass spectrometry provides precision in identifying and characterizing biomolecules associated with viral infections [12,13,14]. It not only facilitates rapid diagnosis and variant detection but also aids in the development of neutralizing antibodies, antiviral agents, and biomarkers for monitoring disease progression [12]. Its potential to uncover novel therapeutic targets makes mass spectrometry a cornerstone of modern virology and personalized medicine [9].

Despite the global vaccination efforts, the long-term impacts of vaccination, particularly on individuals with pre-existing comorbidities, remain insufficiently explored [15,16,17,18,19]. Our study uniquely combines clinical, demographic, and proteomic data to investigate both the impact of COVID-19 infection and the effects of vaccination, with a focus on elderly and multimorbid populations in a clinically well-characterized cohort from the Epidemiological Care Center of the University of Szeged. By integrating clinical data, demographic variables (age and gender), vaccination status, and proteomic analyses, the study provides a nuanced understanding of COVID-19’s impact and the effects of vaccination on at-risk populations. Proteomic profiling of patient sera complements clinical and laboratory findings, offering molecular-level insights into blood samples. Among this cohort, cardiovascular disease (CVD) and hypertension (HT) were the most prevalent comorbidities, emphasizing the need to further examine the effects of vaccination on cardiovascular health in these vulnerable groups.

Additionally, we aimed to identify key biomarkers that may improve diagnostic accuracy and enhance risk stratification. By examining proteomic changes associated with SARS-CoV-2 infection and vaccination, we uncovered molecular signatures that may support the development of personalized treatment strategies and enable the effective monitoring of long-term health outcomes. Addressing the gaps in understanding both infection and vaccination effects, our study underscores the need for a comprehensive, integrated approach. The findings will contribute to future research on emerging infectious diseases and support the broader framework for personalized medicine, guiding more effective public health strategies and pandemic preparedness.

## 2. Results

### 2.1. Clinical Characteristics of Patients

**Figure 1:**
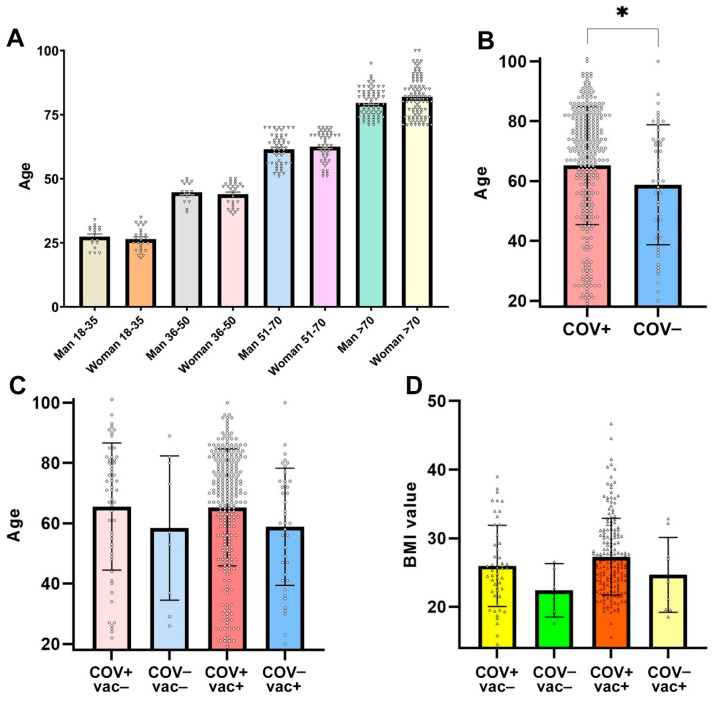
Patient characterization by sex, age, infection/vaccination status, and BMI. (**A**) Sex- and age-stratified distribution of the study population (*n* = 366). Bars represent mean ± SEM. (**B**) COVID-19 status by age distribution. Bars represent mean ± SD. * *p* = 0.0353 (two-tailed independent *t*-test). (**C**) Distribution of COVID-19 (COV+ or COV−) and vaccination status (vac+ or vac−) by age. Bars represent mean ± SD. No statistically significant difference was observed (*p* = 0.19, ANOVA). (**D**) BMI distribution categorized by COVID-19 and vaccination status. Bars represent mean ± SD. No statistically significant difference was observed (*p* = 0.08, ANOVA). Complete descriptive statistics (means, SDs, SEMs, 95% CIs, and *p*-values) underlying panels (**A**–**D**) are provided in Supplementary Table S1.

Blood samples were collected between March 2022 and spring 2023, a period when SARS-CoV-2 infections in Hungary were overwhelmingly caused by Omicron subvariants (BA.1, BA.2, BA.4, and BA.5). Accordingly, the COV+ group primarily represents Omicron-era cases. A total of 366 patients were included, with a mean age of 64.33 ± 19.39 years. Among them, 159 (43.44%) were male and 207 (56.55%) female (Figure 1A). The majority (317 individuals, 86.61%) tested positive for SARS-CoV-2 (COV+), including 133 males and 184 females, while 49 (13.38%) tested negative (COV−), including 26 males and 23 females. Diagnostic testing varied: 299 patients were tested with a rapid test and rapid/quick PCR, nine with a rapid test and PCR, one with PCR only, two with rapid/quick PCR only, and six were confirmed positive by all three methods.

Among the participants, 262 individuals (71.58%) had received at least one COVID-19 vaccine dose (vac+), while 104 (28.42%) were unvaccinated (vac−). The mean interval between the last vaccination and blood sampling was 243.5 ± 7.9 days, confirming that analyses were performed well beyond the acute post-vaccination phase and thus reflect long-term molecular patterns. Within the vaccinated group, 181 individuals (69.08%) received only the BNT162b2 (Pfizer–BioNTech) mRNA vaccine (Table 1). Of these, 120 completed the standard two-dose regimen, 46 received one booster, and 15 received two boosters. Other monovalent vaccinations included 14 individuals (5.34%) who received BBIBP-CorV (Sinopharm), 7 (2.67%) Gam-COVID-Vac (Sputnik V), 6 (2.29%) mRNA-1273 (Moderna), 6 (2.29%) ChAdOx1-S (AstraZeneca), and 1 (0.38%) Ad26.COV2.S (Janssen). In addition, 41 individuals received mixed vaccine regimens (Table 2). Among these, five received at least one dose of mRNA-1273 in combination with ChAdOx1-S (*n* = 2), Ad26.COV2.S (*n* = 1), or BNT162b2 (*n* = 2). One individual received Ad26.COV2.S with BNT162b2, and seven received ChAdOx1-S followed by BNT162b2. A notable subgroup of 16 individuals received BBIBP-CorV along with one or more doses of BNT162b2: 10 completed the two-dose BNT162b2 regimen, 3 received two BNT162b2 boosters, and 2 received three doses. Additionally, 12 individuals received a combination of Gam-COVID-Vac and BNT162b2: 11 completed the standard BNT162b2 regimen, and 1 received the full series with two additional boosters. Vaccine regimen data were unavailable for six individuals (Table 1). Overall, BNT162b2 mRNA vaccination was predominant in this cohort.

Among COV+ individuals, 45 (17 males, 28 females) were in the 18–35 age group, 45 (16 males, 29 females) were aged 36–50 years, 94 (44 males, 50 females) were aged 51–70 years, and 133 (52 males, 81 females) were 70 years or older (Figure 1B). Among vaccinated individuals (vac+), 30 (13 males, 17 females) were aged 18–35, 51 (22 males, 29 females) were aged 36–50, 86 (31 males, 55 females) were aged 51–70, and 95 (39 males, 56 females) were aged 70 years or older (Figure 1C and Supplementary Table S1).

Regarding body mass index (BMI), nine individuals (2.45%) were classified as underweight (BMI < 18.5), 89 (24.31%) as having normal weight (BMI 18.5–24.9), and 142 (38.79%) as overweight (BMI ≥ 25). BMI data were unavailable for 126 individuals, including 106 vaccinated (vac+) participants, precluding BMI classification in these cases. Among COV+ individuals, 8 participants (2 males and 6 females) were underweight, 92 (39 males and 53 females) had normal weight, and 140 (55 males and 85 females) were overweight. Within the vaccinated subgroup, 3 individuals (1 male and 2 females) were underweight, 68 (24 males and 44 females) had normal weight, and 91 (40 males and 51 females) were overweight. BMI values were used solely for descriptive purposes and were not included in inferential statistical analyses.

The cohort was stratified according to both COVID-19 and vaccination status: 222 individuals were COV+ vac+, 95 were COV+ vac−, 40 were COV− vac+, and 9 were COV− vac−. The COV+ vac+ group had an average age of 65.3 years and a mean BMI of 27.3 (Figure 1D and Supplementary Table S1). In the COV+ vac− group, the average age was 58.4 years, with a BMI of 22.4. The COV− vac+ group also had an average age of 58.4 years, with a BMI of 27.3, while the COV− vac− group had an average age of 65.4 years and a BMI of 27.3.

Comorbidities were frequent in the cohort: hypertension (HT) was present in 57.38% of patients, cardiovascular disease (CVD) in 47.54%, diabetes mellitus (DM) in 19.95% (including 5.19% with insulin-dependent DM), chronic lung disease (CLD) in 13.39%, psychiatric and mental disorders (PMD) in 10.56%, and neurological disorders (ND) in 6.83%. Many patients had multiple coexisting conditions. For clarity, acute lung injury (ALI) was analyzed separately, affecting 18.85% of the study population.

In the COV+ vac+ group, the most prevalent comorbidities were HT (59.00%), CVD (51.72%), DM (22.22%), and CLD (13.79%). The same conditions predominated in the COV+ vac− group, with similar proportions: HT (70%), CVD (60%), DM (22.5%), and CLD (12.5%). In the COV− vac+ group, the most common comorbidities were HT (55%), CVD (27.5%), CLD (17.5%), PMD (15%), and DM (12.5%). In the COV− vac− group, frequent comorbidities included HT (66.67%), CVD (44.44%), autoimmune disorders (22.22%), and DM (11.11%). Overall, major comorbidities were similarly distributed across the groups, hence no cohort was substantially healthier or more comorbidity-free than the others.

It is important to note that the cohort consisted of patients under effective medical management. Despite the high prevalence of HT, the average systolic blood pressure in the overall cohort was 133.61 ± 1.17 mmHg and the average diastolic pressure was 79.30 ± 0.70 mmHg. Even among hypertensive individuals, blood pressure remained within the normal range (systolic: 133.93 ± 1.68 mmHg; diastolic: 78.48 ± 0.97 mmHg), indicating adequate antihypertensive therapy. Accordingly, the cohort largely consisted of individuals receiving continuous pharmacological treatment for chronic conditions. The most frequently used medications included antihypertensive agents, lipid-lowering drugs, antiplatelet agents, antidiabetic therapies, as well as diuretics, proton pump inhibitors, and psychotropic medications. In patients with chronic lung diseases, inhaled bronchodilators and corticosteroids were commonly administered. Anticoagulants were used broadly across the cohort, based on established clinical indications.

### 2.2. Risk Assessment

**Figure 2:**
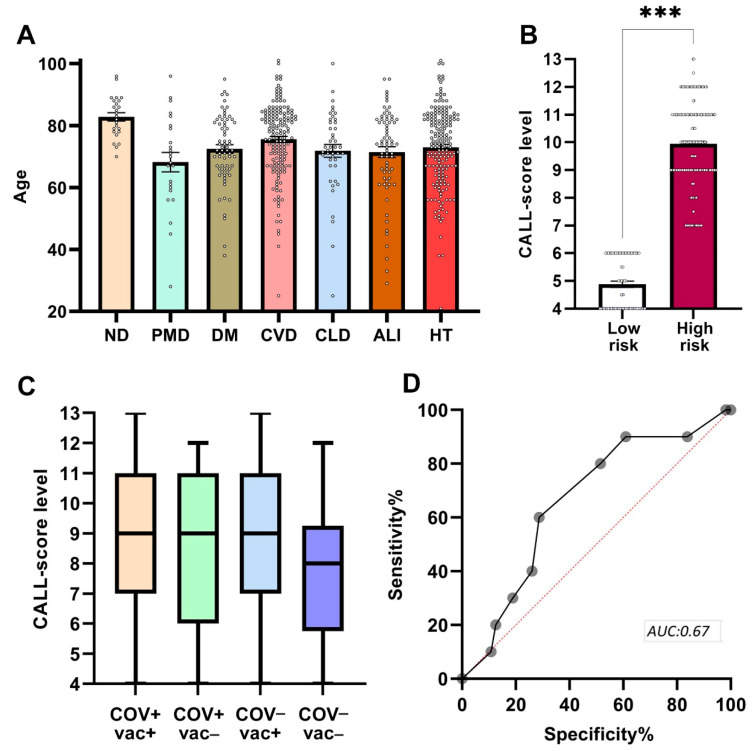
Patient characterization by comorbidities and risk assessment. (**A**) Distribution of comorbidities in the study population (*n* = 366) categorized by age. Bars represent mean ± SEM. (**B**) CALL score stratification among COV+ patients, highlighting a significant difference between groups (independent *t*-test, two-tailed, *** *p* < 0.001). Bars represent mean ± SEM. (**C**) Distribution of CALL scores among the 366 patients based on SARS-CoV-2 infection and vaccination status. Boxes display the median (line) with upper and lower quartiles, and whiskers indicate minimum and maximum values. No significant differences were observed across groups. (**D**) ROC analysis of CALL scores with moderate performance (AUC = 0.67) between COV− vac− and COV+ vac+ groups.

Among the 317 COV+ individuals, comorbidities included HT (57.41%), CVD (50.16%), DM (21.14%), ND (7.57%), and PMD (7.50%). Among the 49 COV− individuals, HT was also the most prevalent (57.14%), followed by CVD (30.61%), CLD (16.33%), PMD (16.33%), DM (12.24%), and ND (4.08%) (Figure 2A).

To assess the risk of severe disease progression, we applied the CALL score, a validated metric that predicts COVID-19 severity based on four parameters: comorbidity, age, lymphocyte count, and LDH levels. The score ranges from 4 (low risk) to 13 (high risk), with higher values reflecting older age, lymphopenia, elevated LDH, and the presence of chronic comorbidities (e.g., cardiovascular, pulmonary, metabolic, or neurodegenerative disorders). This clinically intuitive scoring system supports the identification of patients at elevated risk of clinical deterioration (see Supplementary Table S2).

Among COV+ individuals, 66 patients (20.82%) were classified as low risk, and 251 (79.18%) as high risk. In the COV− group, 10 patients (20.41%) were low risk, and 39 (79.59%) high risk (Figure 2B). The near-identical distribution of CALL risk categories between infected and non-infected groups suggests that the underlying comorbidity burden and systemic vulnerability were comparable, emphasizing the importance of host factors independent of COVID-19 status.

When stratified by vaccination status, 49 vaccinated individuals (18.70%) were classified as low risk, while 213 (81.30%) were high risk. In the unvaccinated group, 27 patients (25.96%) were low risk, and 77 (74.04%) high risk (Figure 2C). This modest difference between vaccinated and unvaccinated groups further supports the interpretation that intrinsic host factors—such as comorbidities, age-related immune decline, and chronic disease load—may play a more dominant role in determining vulnerability than vaccination status alone.

To evaluate the predictive utility of the CALL score, we performed a receiver operating characteristic (ROC) analysis comparing COV− vac− and COV+ vac+ groups. The analysis yielded an area under the curve (AUC) of 0.67, indicating moderate discriminatory performance (Figure 2D). Although the CALL score was originally developed to predict disease progression in COVID-19 patients, we applied it across all patient groups—including those without SARS-CoV-2 infection—as an exploratory composite index of baseline health and systemic physiological stress. This exploratory use is not intended as a validated prognostic application for non-COVID patients but provides a comparative lens for assessing overall clinical burden across the cohort.

### 2.3. SARS-CoV-2 Infection: Comorbidities and Correlations

Pearson correlation analysis identified statistically significant associations between SARS-CoV-2 positivity (COV+) and specific comorbidities, namely CVD (*r* = 0.153, *p* = 0.0032) and ALI (*r* = 0.1708, *p* = 0.001; Table 3). These findings suggest that CVD and ALI might be potential risk factors for SARS-CoV-2 infection in this cohort. However, the low R^2^ values (0.024 and 0.029) indicate weak linear relationships, likely reflecting the cohort’s clinical heterogeneity and the multifactorial nature of COVID-19 susceptibility. To corroborate these associations, Fisher’s exact test was applied to categorical data, confirming a significant relationship between CVD and COV+ status (odds ratio [OR] = 2.63, *p* = 0.005), consistent with the correlation analysis. Although some comorbidities, such as ND (OR = 3.93, *p* = 0.2249) and DM (OR = 2.14, *p* = 0.1629), showed elevated odds ratios, the associations did not reach statistical significance. Similarly, PMD (OR = 0.56), CLD (OR = 0.64), and HT (OR = 1.63) were not significantly associated (Supplementary Table S3).

Correlation analysis of SARS-CoV-2 infection with blood parameters showed a positive correlation with lymphocyte count (*r* = 0.120, *p* = 0.021) and a negative correlation with C-reactive protein (CRP; *r* = −0.114, *p* = 0.029) (Table 4). While statistically significant, the low R^2^ values (0.0145 and 0.013) indicate that these account for only a small proportion of variance and should be interpreted as modest trends rather than strong predictors. The inverse association with CRP likely reflects both elevated CRP in COV− patients with severe comorbidities and suppression of CRP in severe COV+ patients treated with anti-inflammatory agents such as corticosteroids.

**Figure 3:**
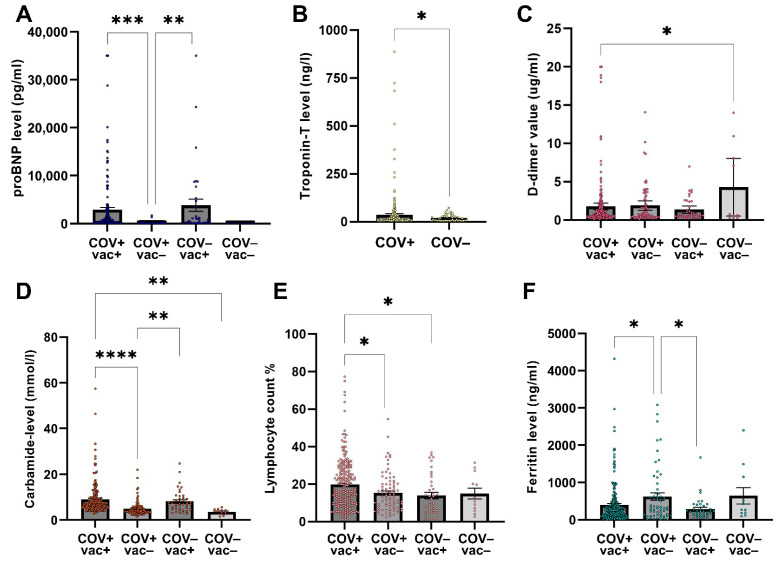
Comparison of blood parameters based on SARS-CoV-2 infection status and vaccination. Bars represent mean ± SEM. (**A**) Plasma proBNP (pg/mL) was higher in COV+ vac+ vs. COV+ vac− (*** *p* < 0.003) and in COV− vac+ vs. COV+ vac− (** *p* < 0.03). (**B**) Serum troponin-T (ng/l) differed between COV+ and COV− (independent *t*-test, * *p* = 0.0485). (**C**) Plasma D-dimer (µg/mL) was significantly higher in COV− vac− than in COV+ vac+ (* *p* = 0.02). (**D**) Serum carbamide (mmol/l) differed between COV+ vac+ and COV+ vac− (**** *p* < 0.0001), COV+ vac− and COV− vac+ (** *p* = 0.0049), and COV+ vac+ and COV− vac− patients (*p* = 0.0060). (**E**) Lymphocyte counts (% of total WBC) were higher in COV+ vac+ vs. COV+ vac− (* *p* = 0.0309) and in COV+ vac+ vs. COV− vac+ (* *p* = 0.0376, ANOVA). (**F**) Serum ferritin (ng/mL) was elevated in COV+ vac+ vs. COV+ vac− (* *p* = 0.0383) and in COV+ vac− vs. COV− vac+ patients (* *p* = 0.0268, ANOVA).

Further analysis revealed intricate relationships between SARS-CoV-2 infection, vaccination status, and key clinical parameters:

Levels of proBNP (N-terminal pro-B-type natriuretic peptide), a biomarker of cardiac stress, differed significantly between COV+ vac+ and COV+ vac− patients (*p* < 0.003), and between COV− vac+ and COV+ vac− patients (*p* < 0.03) (Figure 3A). Notably, the stronger difference between COV+ vac+ and COV+ vac− suggests that vaccination may be a contributing factor.

Troponin-T, a marker of myocardial injury, was significantly higher in COV+ than COV− patients (*p* = 0.0485), consistent with possible myocardial involvement or systemic inflammation associated with SARS-CoV-2 infection (Figure 3B).

D-dimer was significantly higher in COV− vac− patients compared to COV+ vac+ individuals (*p* = 0.02), potentially reflecting a prothrombotic state in the COV− vac− group driven by underlying comorbidities or pre-existing conditions (Figure 3C). Although SARS-CoV-2 infection is typically associated with elevated D-dimer levels, the lower levels in COV+ vac+ individuals may be influenced by the greater anticoagulant use in this group (132 of 261 patients; 50.6%) than in COV− vac− group (1 of 9 patients; 11.1%), which could blunt infection-induced coagulopathy [20,21].

Carbamide showed the largest between-group differences, particularly between COV+ vac+ and COV+ vac− individuals (*p* < 0.0001), between COV+ vac− and COV− vac+ patients (*p* = 0.0049), and between COV+ vac+ and COV− vac− patients (*p* = 0.0060). These elevations may indicate renal impairment or increased protein catabolism, potentially triggered by SARS-CoV-2 infection, immune activation following vaccination, or a combination of both (Figure 3D).

Lymphocyte counts, which are crucial parameters for assessing immune responses, differed significantly between COV+ vac+ and COV+ vac− patients (*p* = 0.0309), and between COV+ vac+ and COV− vac+ patients (*p* = 0.0376, ANOVA) (Figure 3E), possibly reflecting vaccine-induced immune memory, transient redistribution, or individual immune dynamics.

Ferritin levels—an acute-phase reactant and marker of iron storage—differed significantly between COV+ vac+ and COV+ vac− (*p* = 0.0383), and between COV+ vac− and COV− vac+ individuals (*p* = 0.0268, ANOVA), potentially reflecting systemic inflammation, iron dysregulation, or immune activation (Figure 3F). Autoimmune disorders—known to raise ferritin—were more common among the unvaccinated, possibly contributing to these differences [22].

### 2.4. Comorbidity-Driven Deaths in SARS-CoV-2-Positive Elderly Patients

Five patients in the SARS-CoV-2-positive, vaccinated group (COV+ vac+; *n* = 222) died during hospitalization. Despite prior vaccination with BNT162b2—administered on average 230 days before admission, with four patients receiving three doses and one completing the standard two-dose regimen—all tested positive for SARS-CoV-2 via rapid antigen testing during hospitalization. Their deaths were primarily associated with pre-existing severe comorbidities, exacerbated by SARS-CoV-2 infection, rather than predominantly caused by classical COVID-19 pneumonia. These cases are consistent with the complex interaction between advanced age, chronic disease burden, and viral insult.

The deceased patients had a mean age of 82.2 ± 3.7 years and a body mass index of 28.2 ± 1.4 kg/m^2^, consistent with geriatric status and mild obesity. All presented with multiple comorbidities, including HT in all five cases, CVD in four, and type 2 DM in three. Laboratory findings were indicative of systemic deterioration, showing elevated inflammatory markers (CRP: 69.9 ± 22.8 mg/L; IL-6: 166.1 ± 78.5 pg/mL), renal impairment (creatinine: 203.6 ± 56.9 µmol/L; eGFR: 42.0 ± 13.3 mL/min), and significant cardiac stress (proBNP: 16,065 ± 5544 ng/L; troponin T: 189.6 ± 134.1 ng/L). Oxygen saturation averaged 92.9 ± 1.5%, and elevated glucose levels (9.24 ± 2.09 mmol/L) were consistent with hyperglycemia (Supplementary Table S4).

Three of the five patients developed pneumonia, yet only two had radiologically confirmed grade 2 COVID-19 pneumonitis resulting in hypoxic respiratory failure. The third pneumonia case, lacking classical COVID-19 imaging features, was likely of bacterial origin, further complicated by sepsis and acute renal failure requiring hemodialysis. Another patient died of acute cardiac decompensation in the context of bilateral hydrothorax without radiologic signs of pneumonia, indicating a cardiovascular mechanism of death. The fifth patient, with a history of chronic respiratory symptoms, developed subacute-on-chronic respiratory failure following SARS-CoV-2 infection, though no radiologic features of COVID-19 pneumonia were observed.

Despite intensive treatment—including oxygen supplementation, corticosteroids, broad-spectrum antibiotics, antivirals such as remdesivir, and supportive interventions including transfusions and dialysis—all five patients deteriorated rapidly. The absence of classical viral pneumonia in three of the five cases suggests that SARS-CoV-2 infection may have functioned as a precipitating factor that exacerbated underlying chronic disease processes, rather than serving as the primary cause of death. These cases underscore the vulnerability of elderly, multimorbid individuals to adverse outcomes despite vaccination and highlight the importance of personalized, anticipatory care in this population.

In addition to the vaccinated cases, two unvaccinated elderly female patients (aged 91 and 101 years) in the COV+ vac− group (*n* = 95) also died. Both had very low body mass index (18.7 and 19.5 kg/m^2^, group mean ± SEM: 19.13 ± 0.4) and multiple comorbidities, including hypertension, chronic kidney disease (mean creatinine 233 ± 51 µmol/L; eGFR 11 ± 4 mL/min), anemia, and cardiovascular disease. Laboratory findings were notable for marked systemic inflammation (CRP 199.5 ± 162.5 mg/L; IL-6 1106.5 ± 1065.5 pg/mL), neutrophilia (87.45 ± 8.45%) with severe lymphopenia (0.49 ± 0.08 G/L; 9.15 ± 7.45%), elevated proBNP (9227.5 ± 481.5 ng/L) and troponin-T (206.5 ± 120.5 ng/L), and very high procalcitonin levels (27.75 ± 11.1 ng/mL) indicating bacterial co-infection (Supplementary Table S4). Neither case showed radiologic evidence of COVID-19 pneumonia, and causes of death were attributed to cardiorenal failure and sepsis with bacterial co-infection. These observations, together with the overall laboratory profile of the COV+ vac− group, support the interpretation that mortality in this cohort was predominantly linked to advanced age, frailty, and exacerbation of chronic systemic disease rather than viral pneumonia per se.

Importantly, no deaths were recorded among patients in the COV− vac− or COV− vac+ groups.

### 2.5. Vaccination, Comorbidities, and Parameter Correlations

**Figure 4:**
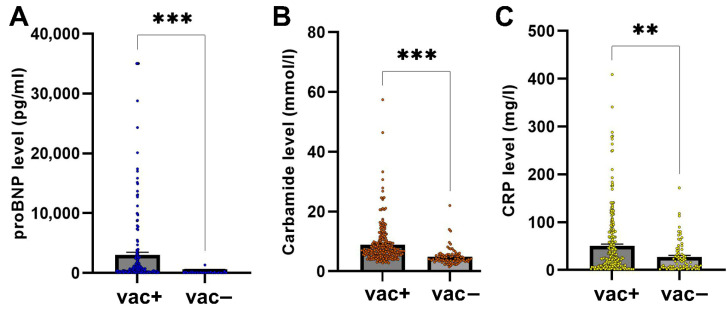
Effect of vaccination on blood parameters. Bars represent mean ± SEM. (**A**) Plasma proBNP levels showed a significant difference between vac+ and vac− patients (independent *t*-test, two-tailed, *** *p* < 0.001). (**B**) Serum carbamide levels were significantly different between vac+ and vac− patients (independent *t*-test, two-tailed, *** *p* < 0.0001). (**C**) Serum CRP levels were significantly different between vac+ and vac− individuals (independent *t*-test, two-tailed, ** *p* = 0.0024).

Among vaccinated patients, 59.54% had HT, 47.71% CVD, 22.52% DM, 16.41% CLD, 9.16% PMD, and 4.96% ND. Laboratory parameters—including proBNP, carbamide, and CRP—were analyzed for correlations with vaccination status. Significant positive correlations were found between vaccination status and proBNP (r = 0.24, *p* < 0.0001), CRP (*r* = 0.113, *p* = 0.034), and carbamide (*r* = 0.301, *p* < 0.0001), suggesting links between vaccination status and markers of cardiac stress (proBNP), inflammation (CRP), and renal function (carbamide) (Figure 4).

To address concerns that CVD may account for higher proBNP levels in vaccinated individuals, we compared blood pressure across groups. No significant differences were found in systolic or diastolic blood pressure between vaccinated and unvaccinated groups (*p* = 0.1205 and *p* = 0.6541, respectively), nor between COV+ and COV− patients (systolic: *p* = 0.9323; diastolic: *p* = 0.2319). Thus, blood pressure does not account for the proBNP differences (Supplementary Figure S1 and Supplementary Table S5). Linear regression analysis revealed that vaccination status was significantly associated with higher proBNP levels (β = 1750, *p* = 0.0103; Table 5). In contrast, no significant association was observed between pre-existing CVD and proBNP levels (β = 654.5, *p* = 0.3179). These findings indicate that the association between vaccination status and proBNP elevation persisted after adjusting for CVD.

Ferritin levels showed a modest but significant negative correlation with vaccination status (*r* = −0.175, *p* < 0.01; Supplementary Table S6), with lower concentrations in vaccinated individuals. Elevated ferritin is commonly associated with autoimmune diseases such as rheumatoid arthritis (RA), systemic lupus erythematosus (SLE), multiple sclerosis, polymyositis, and dermatomyositis [23]. Among unvaccinated participants, seven individuals (6.73%) had one of these conditions: one with polymyositis, two with multiple sclerosis, three with RA, and one with RA–SLE overlap. In contrast, only four of the 262 vaccinated participants (1.53%) had these diagnoses: three with RA and one with polymyositis. The lower ferritin levels in vaccinated individuals may partly reflect the lower prevalence of autoimmune diseases known to elevate ferritin, although differences in immune regulation post-vaccination could also play a role.

### 2.6. Mass Spectrometry

From the full cohort of 366 patients, a representative subset of 64 individuals was selected for mass spectrometry-based proteomic analysis, stratified by SARS-CoV-2 infection and vaccination status, ensuring balanced representation in age, sex, and comorbidities. Of these, 48 had complete clinical and laboratory data, while the remaining 16—although lacking complete laboratory parameters—had documented age, sex, BMI, clinical status, infection history, and vaccination status (8 COV+ and 8 COV−, all unvaccinated). Among the 48 patients with full laboratory datasets, 19 were SARS-CoV-2 negative and unvaccinated (COV− vac−), and 29 were SARS-CoV-2 positive (COV+), including 25 vaccinated (vac+) and 4 unvaccinated (vac−). Of the 25 vaccinated, 92% had spike protein-expressing vaccines—mostly mRNA-based (84%)—and 8% received inactivated vaccines. The mean interval between the last vaccine dose and blood sampling was 214.0 ± 23.4 days (range: 55–490; median: 175 days). Table 6 summarizes the vaccine regimens. Although the cohort included individuals vaccinated with diverse COVID-19 vaccine platforms, the majority received mRNA-based formulations. Given the small number of participants receiving inactivated or viral vector vaccines only, stratified proteomic analysis by vaccine type was not feasible. Therefore, most molecular signatures likely reflect responses to spike protein-expressing vaccines.

**Figure 5:**
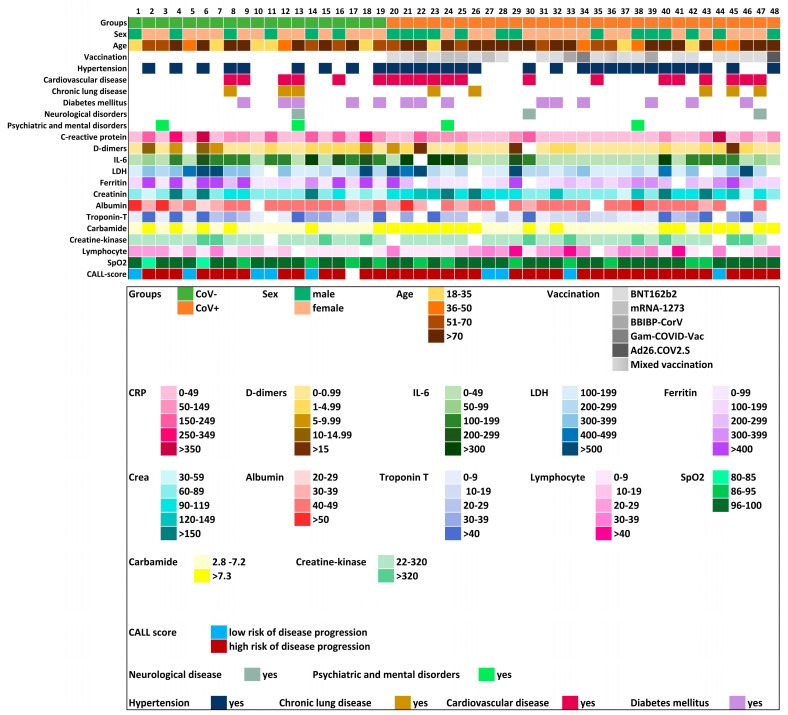
A comprehensive analysis of the patient population: demographic and clinical data. Heatmap of the laboratory parameters of the patients included in the mass spectrometry analysis. The lower table shows the measured values classified according to the color code to which measurement range they belong. Units of blood parameters are provided in Supplementary Table S7.

The cohort of 48 participants with full laboratory datasets included 20 males and 28 females (Figure 5). Regarding age distribution, six participants were aged 18–35 (five in the COV− group and 3 in the COV+ group), 6 were aged 36–50 (2 in the COV− group and 4 in the COV+ group), 12 were aged 51–70 (5 in the COV− group and 5 in the COV+ group), and 24 were aged 70 and above (7 in the COV− group and 17 in the COV+ group). Based on BMI classification, 2 individuals were underweight (both in the COV− group), 8 were of normal weight (3 in the COV− group and 5 in the COV+ group), 18 were overweight (4 in the COV− group and 14 in the COV+ group), and BMI data were unavailable for 20 individuals. Comorbidity data revealed that 21 participants had CVD (7 in the COV− group and 14 in the COV+ group), 8 had CLD (3 in the COV− group and 5 in the COV+ group), 12 had DM (5 in the COV− group and 7 in the COV+ group), and 30 had HT (9 in the COV− group and 21 in the COV+ group). Pearson correlation analysis showed no statistically significant associations between SARS-CoV-2 infection and comorbidities.

The CALL score indicated a low risk of disease progression for 9 individuals (5 in the COV− group and 4 in the COV+ group), while 38 individuals were classified as high risk (13 in the COV− group and 25 in the COV+ group). One individual’s CALL score could not be calculated due to missing data.

Analysis of laboratory blood parameters revealed no significant correlations between SARS-CoV-2 infection and various blood parameters in the 48 patients based on Pearson correlation analysis. Figure 5 shows a heatmap representation of the distribution of blood parameters across patients, visualized using a color scale. (Units of measurement for the laboratory blood parameters are detailed in Supplementary Table S6). Although a full laboratory panel was not available for 16 patients, their samples were still included in the proteomic analyses.

Analysis of protein profile changes across the 64 serum samples detected several proteins significantly differentially expressed between SARS-CoV-2-infected and uninfected individuals. Based on *n* = 64, statistical power exceeded 85% for detecting medium-to-large effect sizes (Cohen’s d ≥ 0.8) at α = 0.05. Correlation analysis identified significant associations with proteins in coagulation, inflammation, and innate immunity (Table 7):

- Coagulation system: fibrinogen alpha (FGA), beta (FGB), and gamma (FGG) chains.
- Inflammatory and immune markers: serum amyloid A1 (SAA1), serum amyloid A4 (SAA4), PGLYRP2, and complement components (C2, C8A)
- Cell surface and extracellular matrix proteins: fibronectin (FN1)

These associations suggest activation of coagulation and innate immune pathways, as well as endothelial involvement in SARS-CoV-2 infection (Supplementary Figures S2–S5).

We also analyzed correlations between protein levels and vaccination status using Pearson correlation. Samples from vaccinated individuals were collected on average 214.0 ± 23.4 days post-vaccination, suggesting long-lasting proteomic patterns. Significant positive correlations were observed for the following proteins (Table 8):

- Fibrinolysis and coagulation system proteins: Coagulation factor V, fibrinogen alpha chain (FGA), fibrinogen beta chain (FGB), fibrinogen gamma chain (FGG), plasminogen (PLG), serpin peptidase inhibitor, clade C (antithrombin (SERPINC1), kininogen-1 (KNG1).
- Complement system and immune response proteins: Complement C1q subcomponent subunit B (C1QB), complement C2 (C2), complement C3 (C3), complement component C8 alpha chain (C8A), complement component (C9), complement factor H-related protein 2 (CFH), complement factor I (CFI), complement factor B (CFB).
- Immune response and acute phase proteins: Serum amyloid A1 (SAA1), serum amyloid A4 (SAA4), peptidoglycan recognition protein 2 (PGLYRP2)
- Heme-binding and immune-related proteins: Haptoglobin (HP), hemopexin (HPX), apolipoprotein E (APOE), carboxypeptidase N subunit 2 (CPN2), carbonic anhydrase 1 (CA1).
- Extracellular matrix and cell surface proteins: Fibronectin (FN1), vitronectin (VTN), C-type lectin domain family 3 member B (CLEC3B).

Our mass spectrometry data point to distinct molecular response profiles between SARS-CoV-2 infection and long-term post-vaccination status (Table 9):

- Coagulation activation markers, particularly fibrinogen chains (FGA, FGB, FGG), were positively correlated in both groups, but with significantly stronger associations in the vaccinated group (e.g., FGA: *r* = 0.6126, *p* < 0.0001 vs. r = 0.2888, *p* < 0.01 in infected). As central components of the coagulation cascade and acute-phase reactants, these proteins are linked to both thrombotic and inflammatory activation. The stronger association post-vaccination may be compatible with sustained endothelial activation or vascular remodeling. Notably, SERPINC1 correlated only in vaccinated individuals (*r* = 0.454, *p* < 0.0001), which could reflect a longer-term compensatory anticoagulant response.
- Acute-phase proteins (SAA1, SAA4) were elevated in both groups, but significantly higher in vaccinated individuals, particularly SAA4 (*r* = 0.7051, *p* < 0.0001). Oxidative stress markers (HP, HPX) were also exclusively correlated in vaccinated individuals, which may be compatible with persistent low-grade inflammation and redox imbalance more than six months post-vaccination.
- Complement activation was present in both groups (C2, C8A), but amplified in the vaccinated, with additional exclusive correlations for CFB, CFH, CFI, and C1QB, compatible with a broad, long-lasting complement cascade activation, including both effector and regulatory components.
- Extracellular matrix remodeling proteins (FN1, CLEC3B, VTN) showed stronger correlations in the vaccinated group, which could be compatible with ongoing endothelial remodeling and possible microvascular changes six months after immunization.
- Neutrophil-mediated innate immune activation, including FCGR3B (component of neutrophil extracellular traps) and PGLYRP2 (microbial recognition), was exclusively observed in the infected group, consistent with an acute-phase, pathogen-driven immune response distinct from the post-vaccination profile.
- Finally, APOE, associated with lipid metabolism and neuroinflammation, was only correlated in vaccinated individuals (*r* = 0.3238, *p* < 0.01), a pattern that could be compatible with long-term immunometabolic changes or increased neuroinflammatory susceptibility.

The proteomic profiles are consistent with acute-phase innate immune activation during SARS-CoV-2 infection, whereas vaccination is associated with persistent, low-grade proinflammatory, procoagulant, and tissue remodeling processes detectable more than six months post-vaccination. These findings could inform our understanding of long-term vaccine-associated immunological states and their relevance in vulnerable populations.

## 3. Discussion

Our study provides an integrated clinical, biochemical, and proteomic characterization of a diverse patient cohort with varying SARS-CoV-2 infection and vaccination histories, revealing distinct and persistent pathophysiological signatures. By combining mass spectrometry profiling with detailed clinical datasets, we observed biologically and clinically relevant alterations associated with both acute infection and long-term post-vaccination status. As an observational study, these findings warrant cautious interpretation but reveal a complex interplay between demographics, comorbidities, biomarkers, and molecular changes, offering new insights into the systemic effects of viral exposure and immunization. In particular, our data suggest that SARS-CoV-2 infection and vaccination—especially with mRNA-based COVID-19 vaccines—are associated with distinct patterns of inflammation, coagulation, and organ function in elderly and multimorbid individuals.

The cohort composition reflects the demographic and clinical complexity typically seen in internal medicine. With a mean age over 64 years and a high prevalence of chronic conditions—particularly HT and CVD—participants were inherently at increased risk for infection-related complications and altered immune responses. The high proportion of SARS-CoV-2-positive individuals (86.6%) reflects hospital-based testing during pandemic peaks, while the substantial representation of vaccinated participants (82.2%), most of whom received spike-expressing vaccines (predominantly mRNA), allowed evaluation of long-term immunological effects. The average interval of over 240 days between vaccination and blood sampling reduces confounding by acute post-vaccination responses and supports interpretation of sustained molecular changes.

Comorbidity profiles were balanced across infected and non-infected as well as vaccinated and unvaccinated subgroups, suggesting that the observed clinical, proteomic, and biochemical differences are unlikely to be driven by baseline health disparities. Blood pressure was well controlled in most patients, minimizing confounding in interpreting cardiac stress markers such as proBNP. Although BMI data were missing in 34.4% of patients, BMI was not included in inferential analyses and thus does not affect the study’s core findings—though this gap should still be considered.

The CALL score identified a high-risk status in the majority of individuals regardless of infection or vaccination status [24]. This supports the concept that multimorbidity and systemic frailty—rather than viral status alone—are dominant drivers of physiological vulnerability. Although the CALL score showed moderate discriminatory performance (AUC = 0.67), it appeared insensitive to vaccination status, suggesting that updated risk tools incorporating immunization history, biological age, and immune remodeling may be warranted. Here, the CALL score was also applied to non-COVID patients as an exploratory, hypothesis-generating composite index reflecting general physiological stress and comorbidity burden, given that its components—age, lymphocyte count, and LDH—are relevant beyond SARS-CoV-2 infection. To preserve a consistent statistical framework, CALL scores were calculated across all four patient groups (COV+ vac+, COV+ vac−, COV− vac+, and COV− vac−), including non-infected individuals. While originally validated in infected patients, its use here in non-infected groups was exploratory and should not be interpreted as predictive, but rather as a descriptive frailty-related measure.

SARS-CoV-2 infection was significantly associated with ALI and CVD, reflecting the systemic impact of the virus in clinically vulnerable populations. Several laboratory parameters—particularly lymphocyte count and C-reactive protein (CRP)—showed counterintuitive associations with infection status, illustrating the complexity of interpreting inflammatory biomarkers in medically managed settings. While statistically significant, these correlations had low R^2^ values (<0.03), indicating weak linear relationships likely shaped by multifactorial influences, including treatment timing, comorbidities, and disease trajectory. For instance, the positive correlation between lymphocyte count and SARS-CoV-2 positivity may reflect immune reactivation during recovery or milder disease stages [25]. Conversely, the inverse correlation between CRP and infection status is likely explained by corticosteroid and anti-inflammatory use, which suppress acute-phase reactants. Widespread prophylactic or therapeutic heparin administration likely contributed to stable D-dimer levels, despite proteomic evidence of ongoing coagulation activation [26]. These examples reinforce the need to interpret biomarker changes in light of standardized medical interventions.

Among the clearest signals, elevated proBNP levels were consistently associated with vaccination, even at a mean post-vaccination interval of over 240 days. ProBNP is a recognized biomarker of cardiac stress, routinely used to assess heart failure and thromboembolic risk [27,28,29,30]. Elevated proBNP has also been linked to increased mortality in COVID-19, independent of baseline cardiac conditions [31,32,33]. In our cohort, vaccinated individuals—predominantly mRNA vaccine recipients—displayed sustained proBNP elevation, consistent with chronic, low-grade myocardial stress. This aligns with prior reports of post-vaccination cardiac complications, including myocarditis and pericarditis, especially following mRNA vaccination [34,35]. Persistence well beyond expected immune activation windows is consistent with ongoing subclinical inflammation, endothelial dysfunction, or immune-mediated cardiac strain, possibly linked to repeated spike protein exposure [36,37,38,39]. Notably, linear regression analysis showed that proBNP elevation was significantly associated with vaccination status (β = 1750, *p* = 0.0103), but not with pre-existing CVD (β = 654.5, *p* = 0.3179), indicating that the observed elevation in vaccinated individuals is unlikely to be driven solely by underlying heart conditions. While echocardiographic or direct cardiologic assessments were not available, elevated proBNP here should be considered an indirect indicator of possible cardiac stress—warranting follow-up studies, especially in mRNA-vaccinated individuals with pre-existing cardiovascular conditions [40].

Carbamide levels were also significantly higher in vaccinated SARS-CoV-2-positive individuals (*p* < 0.0001), consistent with renal stress or heightened protein catabolism from combined effects of infection and vaccination. While impaired renal function is known to elevate proBNP levels, this explanation appears unlikely to fully account for our findings [41,42]. Renal disease prevalence was actually higher among unvaccinated individuals (11.71%) than vaccinated ones (7.63%), making renal dysfunction an unlikely sole explanation for both carbamide and proBNP patterns. This further supports the possibility of a vaccination-associated component and highlights the need for longitudinal studies to evaluate renal and cardiac effects of repeated spike protein exposure.

Interestingly, while ferritin levels were elevated in individuals infected with SARS-CoV-2, they were significantly reduced in vaccinated patients. This pattern may indicate that vaccination may attenuate the overall inflammatory response and provide protection against excessive iron dysregulation and cytokine-mediated tissue damage—both hallmarks of severe COVID-19 [43]. However, comorbidities can influence ferritin levels independently of infection or vaccination. Autoimmune diseases such as RA, SLE, multiple sclerosis, polymyositis, and dermatomyositis are known to induce hyperferritinemia [44]. In our cohort, these conditions were more prevalent among unvaccinated individuals (22.22% in COV− vac−, 5.26% in COV+ vac−) compared to vaccinated groups (0% in COV− vac+, 1.8% in COV+ vac+), likely contributing to the observed ferritin differences.

Mass spectrometry analysis supported these clinical observations, revealing differential expression of coagulation factors (fibrinogen chains), acute-phase proteins (SAA1, SAA4), complement components (C2, C8A), and immune-related proteins (FCGR3B, CLEC3B). Although several vaccine types were represented, most participants had received mRNA-based or other spike protein-expressing vaccines. Given the limited size of non-mRNA subgroups—particularly those vaccinated with the inactivated platform—vaccine-specific proteomic comparisons were underpowered and therefore not performed. Consequently, the proteomic profile in vaccinated individuals is likely shaped by the long-term systemic effects of spike-expressing vaccine platforms in real-world mRNA-dominant settings. Notably, several proteomic changes mapped closely to the functional pathways implicated by the clinical biomarkers, reinforcing the plausibility of sustained cardiac, vascular, and inflammatory stress in the post-vaccination state. The mass spectrometry-based proteomic analysis also enabled a molecular-level comparison between acute SARS-CoV-2 infection and long-term post-vaccination states, indicating differences in immunological profiles. In SARS-CoV-2-positive patients, proteomic changes were consistent with reported features of COVID-19 pathophysiology, including neutrophil activation (e.g., FCGR3B, PGLYRP2), moderate coagulation and acute-phase responses (FGA, FGB, SAA1/4), and early complement cascade activation (C2, C8A), consistent with classical innate immune engagement and endothelial injury [45,46,47,48,49,50,51,52]. In contrast, the proteomic signature observed in vaccinated individuals—despite an average of 214 days since their last vaccine dose—is consistent with the possibility of persistent immune alterations. These individuals exhibited markedly elevated levels of all three fibrinogen chains (FGA, FGB, FGG), alongside increased expression of the anticoagulant SERPINC1, indicating a hypercoagulable state with concurrent anticoagulant activity, consistent with endothelial stress or subclinical coagulopathy [53,54,55]. Persistent upregulation of acute-phase reactants such as SAA1, SAA4, haptoglobin (HP), and hemopexin (HPX) points to chronic low-grade inflammation extending well beyond the expected duration of post-vaccination immune activation [56,57,58]. Furthermore, a broad activation pattern of the complement system—encompassing both effector components (e.g., C2, C3, C8A, C9) and regulatory proteins (e.g., CFH, CFI, C1QB)—is consistent with prolonged innate immune stimulation in the absence of active infection [59]. Elevated levels of extracellular matrix remodeling proteins, including fibronectin (FN1), tetranectin (CLEC3B), and vitronectin (VTN), further imply that endothelial and vascular remodeling may persist in the post-vaccination setting [60]. Notably, the absence of neutrophil-associated markers, which were prominent in the infected group, supports the interpretation that this sustained systemic signature is not the result of acute infection but more likely reflects persistent immune alterations triggered by the vaccine. Of particular interest is the selective upregulation of APOE—a molecule involved in lipid metabolism, vascular homeostasis, and neuroinflammatory signaling—which warrants further investigation as a potential marker of metabolic or neuroimmune changes after repeated genetic-based immunization. This close alignment between the laboratory biomarker trends and proteomic signatures strengthens the argument that the observed changes are not random findings, but rather interconnected manifestations of persistent post-vaccination physiological remodeling. While these findings are unlikely to represent only benign residuals of vaccine-induced immune memory, they remain associations and require confirmation in longitudinal studies. Rather, the presence of proinflammatory, prothrombotic, and vascular remodeling biomarkers seven months (214 days) post-vaccination is consistent with the possibility that the systemic effects of spike-expressing, predominantly mRNA-based vaccines may be more prolonged and biologically significant than previously assumed. This is particularly relevant for elderly and multimorbid individuals, who may be disproportionately impacted by sustained molecular responses following repeated vaccination. Although the cross-sectional observational design limits the ability to establish causality, our proteomic findings underscore the need for focused longitudinal studies to evaluate the long-term effects of mRNA vaccine platforms—particularly concerning chronic inflammation, coagulation risk, endothelial function, and systemic resilience in vulnerable populations.

These findings collectively suggest that both SARS-CoV-2 infection and vaccination can induce measurable alterations across multiple physiological systems. The observed changes in inflammation, coagulation, immune activation, and organ function carry important implications for personalized risk assessment, particularly in individuals with pre-existing comorbidities.

Despite its strengths, this study has several important limitations. Its observational, single-center, cross-sectional design restricts generalizability and allows only the identification of associations rather than causal inference. It also remains unclear whether the observed biomarker differences were present before infection or vaccination. To mitigate these constraints, we applied strict inclusion criteria, standardized sampling and laboratory protocols, and adjusted for key confounders in the statistical models. Subgroups were established with evenly distributed comorbidities, ensuring that no group was systematically healthier than another. Most participants were under effective medical management for chronic conditions, supporting the conclusion that pre-existing comorbidities did not disproportionately influence outcomes.

Another limitation is the small size of the COV− vac− group (*n* = 9), which reduces the robustness of comparisons involving this subgroup. These patients were nevertheless retained as they provided important clinical context, having presented with COVID-like symptoms despite negative SARS-CoV-2 tests, and often carried severe comorbidities.

The cohort included both hospitalized and ambulatory individuals, enhancing the real-world relevance of the findings. However, asymptomatic and otherwise healthy individuals without comorbidities were not available for inclusion in such clinical settings. Thus, the selected control groups reflect the practical spectrum of patients encountered during the pandemic.

Subgroup analysis by vaccine platform was constrained by small sample sizes and widespread heterologous vaccination. The majority of patients had received mRNA-based regimens, while inactivated vaccines were uncommon. Although this precluded detailed platform-specific comparisons, the predominance of mRNA regimens makes the findings most applicable to such vaccination strategies.

To reduce variability from viral heterogeneity, recruitment was restricted to March 2022–spring 2023, when Omicron subvariants BA.1, BA.2, BA.4, and BA.5 were predominant in Hungary. This provided a consistent viral background for analysis. Blood samples were also collected well beyond the acute post-vaccination period, allowing assessment of more durable effects.

Finally, as a cross-sectional study, our analysis captures only a single time point. Nevertheless, the consistent elevation of specific biomarkers—observed on average 243.5 days after vaccination—suggests sustained physiological alterations that merit prospective, longitudinal investigation, particularly in clinically vulnerable populations.

Future research should include standardized treatment data, control for medication use, and apply longitudinal, multicenter designs. Such efforts are needed to assess the persistence, reversibility, and clinical implications of the molecular changes observed in association with SARS-CoV-2 infection and vaccination.

## 4. Materials and Methods

### 4.1. Study Population and Data Collection

This study was approved by the Ethics Committees of the Regional and Institutional Review Board of the University of Szeged (Approval Number: 23/2022-SZTE RKEB, Approval date: 21 February 2022). The Regional Human Biomedical Science and Research Ethics Committee evaluated the biomedical research plan from both ethical and medical perspectives, concluding that it complies with the provisions of Decree 23/2002 of the Ministry of Health and Government Decree 235/2009 (X.20.). The study was conducted following the principles outlined in the Declaration of Helsinki. All participants provided written informed consent prior to inclusion, and data were collected in an anonymized format.

Comprehensive patient data were gathered, focusing on key parameters, including COVID-19 status (positive or negative: COV+ or COV−), vaccination status, age, sex, and body weight. COV− vac− individuals were selected from hospitalized patients confirmed to have no history of SARS-CoV-2 infection or vaccination, based on medical records and patient history. Additionally, information on various health conditions was recorded, such as HT, CVD, chronic lung disease (CLD), neurological disorder (ND), psychiatric and mental disorder (PMD), and other comorbidities, including diabetes mellitus (DM). Blood profile data, including various hematological parameters, were also collected. The blood sample collection in our cohort took place between March 2022 and spring 2023 at the Epidemiological Care Center, Department of Internal Medicine, Faculty of Medicine, University of Szeged. During this period, the predominant circulating SARS-CoV-2 variants in Hungary were the Omicron subvariants BA.1, BA.2, BA.4, and BA.5, as reported by national public health data. This approach provided a robust foundation for the subsequent analyses. Notably, the interval between vaccination and blood sampling was 243.5 ± 7.9 days (median: 227.5 days; *n* = 226), allowing the assessment of vaccine-related effects beyond the acute post-vaccination phase.

To ensure data accuracy and reliability, all entries underwent rigorous cross-checking and validation by at least two independent researchers. This careful process strengthened the reliability of the data.

### 4.2. Blood Sample Preparation

Venous blood samples were collected from patients during routine hospital procedures using 6 mL VACUETTE^®^ K3EDTA Blood Collection Tubes (cat. no.: 454021; Greiner Bio-One, Austria). COVID-19 patients received standard treatment protocols, including anticoagulants, and blood samples for both routine laboratory testing and proteomic analyses were collected after therapy initiation, ensuring that measured biomarkers reflected disease-related changes during the acute clinical phase. Some of the collected samples were immediately analyzed for clinical parameters, while the remaining samples were processed according to the manufacturer’s protocol (Thermo Fisher, Waltham, MA, USA) [61,62]. The processed plasma/serum was stored at −20 °C until further analysis. All samples underwent a single freeze–thaw cycle before analysis.

Extensive data collection accompanied blood sampling, including parameters such as SARS-CoV-2 infection status (COV+ or COV−), vaccination status, age, sex, body weight, and associated health conditions. Documented comorbidities included HT, CVD, CLD, and other related conditions. Blood profiles, including various hematological parameters, were also recorded.

To ensure accuracy and reliability, all collected data were independently validated by at least two researchers.

### 4.3. CALL Score

The CALL score, developed by Ji et al., is a predictive model designed to assess the risk of COVID-19 progression with high sensitivity and specificity [63]. This scoring system evaluates clinical deterioration based on four factors: Comorbidity (C), Age (A), Lymphocyte count (L), and Lactate Dehydrogenase (LDH) [24]. Relevant comorbidities include neurodegenerative diseases CLD, CVD, DM, and HT. The formula for the CALL score is as follows:

CALL score = Comorbidity factor + Age factor + Lymphocyte factor + LDH factor [24]. The score ranges from 4 (indicating minimal risk) to 13 (indicating high risk), determined as follows:

- Comorbidity: Presence of one or more comorbidities increases the risk score.
- Age factor:
  o 3 points if the patient is over 60 years old.
  o 1 point if the patient is 60 years old or younger.
- Lymphocyte factor:
  o 3 points if the absolute lymphocyte count is ≤1 × 10^9^/L.
  o 1 point if the absolute lymphocyte count is >1 × 10^9^/L.
- LDH factor:
  o 3 points if LDH >500 U/L.
  o 2 points if LDH is between 250 and 500 U/L.
  o 1 point if LDH <250 U/L.

The CALL score provides a straightforward and reliable method for identifying patients at high risk of clinical deterioration. Supplementary Table S2 summarizes the details of these parameters.

### 4.4. Mass Spectrometry Analysis of Patient Sera

From the full cohort of 366 patients, a representative subset of 64 individuals was selected for mass spectrometry (MS)-based proteomic analysis to investigate molecular changes associated with SARS-CoV-2 infection and COVID-19 vaccination. Stratified random selection was performed based on infection status (COV+ vs. COV−) and vaccination status (vac+ vs. vac−), ensuring diversity in age, sex, and comorbidity profiles. Of these 64 individuals, 48 had complete clinical and laboratory data and were included in integrated clinical–proteomic analyses. This subgroup comprised 29 COV+ individuals (25 vaccinated, 4 unvaccinated) and 19 COV− individuals (all unvaccinated). The COV− vac− group was selected solely based on the absence of SARS-CoV-2 infection and vaccination, without excluding individuals based on comorbidities. The remaining 16 individuals (8 COV+ vac− and 8 COV− vac−) had limited clinical data and were included only in group-specific MS analyses.

For each sample, 1 µL of human serum was denatured with GuHCl and reduced with DTT (5 µL of 25 mM ABC + 6 M GuHCl + 10 mM DTT) for 30 min at room temperature. Alkylation was performed with IAM (35 µL of 25 mM ABC + 22 mM IAM + 0.1 µg trypsin), followed by digestion with trypsin at 47 °C for 3 h. After digestion, 150 µL of 0.1% FA and 8 µL of Evosep were added to the mixture. The digestion mixtures were acidified, and one-eighth of each total digestion volume was loaded onto a single-use trapping mini-columns (Evotip, 1/8 of the samples). Each sample was analyzed in two technical replicates using a data-dependent LC-MS/MS method.

The analyses were performed on an Evosep One system (LC: 30 SPD; MS1: R = 120,000) coupled to a linear ion trap–Orbitrap mass spectrometer (Orbitrap-Fusion Lumos, Thermo Fisher Scientific) operating in positive ion mode. Data acquisition followed a data-dependent strategy, where multiply charged ions were selected in cycle-time from each MS survey scan for ion-trap HCD fragmentation. MS spectra were acquired in the Orbitrap (R = 60,000), and MS/MS data were collected in the ion trap [64]. Raw data were converted into peak lists using the in-house Proteome Discoverer (v1.4). Technical replicates were combined and searched against the SwissProt HUMAN protein database (downloaded on 20 July 2022, containing 51,961 proteins) using the in-cloud Protein Prospector search engine (v5.15.1). The search parameters included trypsin as the enzyme, allowing up to two missed cleavages, with mass accuracies set to 5 ppm for precursor ions and 0.6 Da for fragment ions (both monoisotopic). Fixed modifications included carbamidomethylation of cysteine residues, while variable modifications included acetylation of protein N-termini, methionine oxidation, and cyclization of N-terminal glutamine residues, allowing a maximum of two variable modifications per peptide. Identification criteria required minimum scores of 22 and 15, and maximum E values of 0.01 and 0.05 for protein and peptide identifications, respectively [64,65].

All serum samples were included in meta-analyses using a modified spectral counting approach (relative peptide count × deltaCoverage%) for label-free quantification [65]. This method was conceptually adapted from the label-free proteomic strategy described by Branson and Freitas but includes a novel adjustment that integrates both peptide spectral counts and protein sequence coverage [66]. Specifically, for each identified protein, we calculated a relative peptide count (i.e., the number of unique peptides per protein, normalized across all samples) and multiplied it by the deltaCoverage%, which reflects the difference in sequence coverage between experimental groups. The resulting value served as a relative abundance score, enhancing sensitivity and reducing bias in protein quantification compared to spectral counting alone. This adjustment improves detection of biologically relevant protein changes in complex matrices such as serum. The remaining 16 samples, for which only SARS-CoV-2 status was known, were excluded from meta-analyses. A significant increase in protein abundance in COV+ samples was defined as the detection of a protein in the sera of at least three patients, with at least five unique peptides, higher relative peptide counts, and greater protein coverage compared to the COV− group. Statistical analysis of protein abundance differences between groups was performed using one-way ANOVA, with *p* < 0.05 considered significantly differentially abundant.

### 4.5. Statistical Analysis

Statistical analyses were performed using IBM SPSS version 24.0 (IBM Corp, Armonk, NY, USA). Differences between the two groups were assessed using an independent sample *t*-test. For multiple comparisons, one-way analysis of variance (ANOVA) with Sidak post hoc analysis was applied. Pearson correlation was used to investigate the relationships between COVID-19, various diseases, and blood parameters. Additional tests were conducted to assess the effects of vaccination. Descriptive statistics were employed to analyze comorbidity data among the study participants. Graphs were created in GraphPad Prism version 10.1.2, with data presented as mean ± standard deviation (SD) or standard error of the mean (SEM) on scatter plots. A *p*-value < 0.05 was considered statistically significant for all tests.

In the analysis of protein profile changes across the 64 serum samples, we identified several proteins that were significantly differentially expressed between SARS-CoV-2-infected and uninfected individuals. Based on this sample size (*n* = 64), the estimated statistical power exceeded 85% for detecting medium-to-large effect sizes (Cohen’s d ≥ 0.8) at a significance level of α = 0.05, ensuring adequate sensitivity to detect biologically meaningful differences. ANOVA revealed significant overall differences in selected proteins across the infection/vaccination subgroups, and post hoc Tukey tests confirmed that these differences remained statistically significant for specific pairwise comparisons.

## 5. Conclusions

This study demonstrates that both SARS-CoV-2 infection and spike protein-expressing COVID-19 vaccination are associated with distinct systemic alterations at the clinical, biochemical, and proteomic levels. Acute infection was linked to classical inflammatory and innate immune activation, whereas vaccinated individuals—sampled on average 210+ days after their last, predominantly spike-expressing vaccine dose—exhibited a molecular profile suggestive of low-grade inflammation, procoagulant activity, complement activation, and vascular remodeling. These post-vaccination associations were detectable across diverse clinical backgrounds and occurred independently of acute illness.

Although largely subclinical, the reproducible presence of these signatures raises important questions regarding long-term immune imprinting, endothelial function, and potential cumulative physiological burden in elderly and multimorbid individuals. These findings underscore the value of molecular biomarkers in personalized risk assessment and highlight the importance of longitudinal studies to clarify the persistence, reversibility, and clinical significance of such changes.

Future large-scale, longitudinal studies are warranted to validate these findings, assess the clinical significance of the identified proteomic markers, and determine the persistence and reversibility of vaccine-induced molecular changes. Such efforts are critical for optimizing immunization strategies, enhancing patient safety, and advancing translational applications in personalized medicine and public health.

## Figures and Tables

**Table 1:** COVID-19 vaccine types, dosing, and age demographics among the vaccinated (vac+).

Vaccine Type	n	Age (Mean ± SEM)	Comments
BNT162b2	181	66.23 ± 1.47	120 received the standard 2-dose regimen 46 received one booster, and 15 received two boosters
BBIBP-CorV	14	43.43 ± 3.47	All received the standard two-dose regimen
Ad26.CoV2.S	1	88.00	Single-dose regimen
Gam-COVID-Vac	7	55.00 ± 6.68	6 received the standard two-dose regimen; 1 received an additional booster
mRNA-1273	6	67.83 ± 6.32	2 received the standard two-dose regimen; 4 received one booster
ChAdOx1-S	6	44.67 ± 6.22	All received the standard two-dose regimen
Mixed Regimens	41	65.49 ± 2.32	All received at least one mRNA vaccine dose (mRNA-1273 or BNT162b2)
Unknown	6	65.28 ± 3.14	Vaccine type information unavailable

**Table 2:** Mixed vaccine regimens.

Mixed Vaccine Regimen	n
mRNA-1273 + other (BNT162b2/ChAdOx1-S/Ad26.CoV2.S)	5
Ad26.CoV2.S + BNT162b2	1
ChAdOx1-S + BNT162b2	7
BBIBP-CorV + BNT162b2	16
Gam-COVID-Vac + BNT162b2	12

**Table 3:** Correlation between COVID-19 and CVD or ALI.

	CVD	ALI
Correlation Coefficient (*r*)	0.154	0.171
95% Confidence Interval	0.052 to 0.252	0.070 to 0.269
R^2^	0.024	0.029
*p* (two-tailed)	0.003	0.001
Number of Pairs	366	366

**Table 4:** Correlation analysis between COV+ and lymphocyte count or CRP.

	Lymphocyte Count	CRP
r	0.121	−0.114
95% Confidence Interval	0.018 to 0.220	−0.214 to −0.012
R squared	0.0145	0.013
*p* (two-tailed)	0.021	0.029
Number of XY Pairs	366	366

**Table 5:** Regression analysis of proBNP by vaccination and CVD.

Variable	β	Standard Error	95% CI	*p*-Value
Vaccination	1750	678.3	415.8–3085	0.0103
CVD	654.5	654.3	−632.9–1942	0.3179

**Table 6:** Vaccine regimens in the mass spectrometry proteomic subcohort.

Vaccine Regimen	n	%
BNT162b2 (Pfizer-BioNTech) only	16	64.0
−2 doses (no booster)	4	16.0
−2 doses + 1 booster	8	32.0
−2 doses + 2 boosters	4	16.0
mRNA-1273 (Moderna) only (3 doses)	4	16.0
BBIBP-CorV (Sinopharm) only	2	8.0
Gam-COVID-Vac (Sputnik) only	1	4.0
Ad26.COV2.S (Janssen) only	1	4.0
Heterologous (2 × BBIBP-CorV + 2 × BNT162b2)	1	4.0

**Table 7:** Correlation of plasma proteins with COVID-19.

Protein Name	*p*-Value	r-Value
FN1	0.0288	0.2173
FGA	0.0068	0.2888
SAA1	0.0217	0.2173
SAA4	0.0083	0.2769
FGB	0.0292	0.1984
FGG	0.0381	0.1812
PGLYRP2	0.0221	0.216
CLEC3B	0.0201	0.2221
FCGR3B	0.0019	0.3612
C2	0.0087	0.2736
C8A	0.0076	0.2816

This table presents Pearson *r*-values and corresponding *p*-values, highlighting significant positive correlations with COVID-19 positivity.

**Table 8:** Correlation of plasma proteins with COVID-19 vaccination.

Protein Name	*p*-Value	r-Value
FN1	0.0041	0.3445
FGA	<0.0001	0.6126
HP	0.0436	0.1725
HPX	0.0027	0.3407
APOE	0.0037	0.3238
SAA1	0.0005	0.4315
SAA4	<0.0001	0.7051
CFH	0.0179	0.2293
CFB	<0.0001	0.6555
CFI	0.0032	0.332
PLG	0.0006	0.4226
VTN	0.0237	0.2117
FGB	0.0003	0.4493
FGG	0.0033	0.3307
PGLYRP2	0.0019	0.3614
C3	0.0007	0.4127
F5	0.0054	0.3016
CLEC3B	0.0039	0.3207
SERPINC1	0.0003	0.454
KNG1	0.0015	0.3742
C1QB	0.0074	0.2837
C2	<0.0001	0.6228
C8A	<0.0001	0.5666
C9	0.0033	0.331
CA1	0.046	0.1689
CPN2	0.009	0.2716

This table presents *r*- and *p*-values for vaccination-related correlations. Significant positive associations are consistent with a sustained proinflammatory, prothrombotic, and tissue remodeling signature in vaccinated individuals.

**Table 9:** Proteomic pathway activation between SARS-CoV-2 infection and vaccination.

Biological Process	Acute Infection	Long-Term Vaccination
Coagulation activation	Moderate	Strong (FGA, FGB, FGG, SERPINC1↑)
Acute-phase response	Present (SAA1/4↑)	Strong (SAA1/4↑↑, HP, HPX)
Oxidative stress	Not detected	Elevated (HP, HPX↑)
Complement activation	Partial (C2, C8A)	Broad (C2, C8A, CFB, CFH, CFI, C1QB↑)
ECM remodeling/ Endothelial damage	Moderate (FN1, CLEC3B)	Enhanced (CLEC3B, VTN↑)
Neutrophil response	Present (FCGR3B, PGLYRP2)	Absent
Lipid metabolism/ neuroinflammation	Not detected	APOE↑

Summary of biological processes and associated plasma protein changes identified via proteomic correlation analysis in patients with acute SARS-CoV-2 infection versus those evaluated long after COVID-19 vaccination. Stronger activation of coagulation, complement, oxidative stress, and ECM remodeling pathways is observed post-vaccination, while acute infection is characterized by prominent neutrophil and innate immune responses.

## Data Availability

Data are contained within the article or Supplementary Materials.

## Supplementary Materials

The following supporting information can be downloaded at: https://www.mdpi.com/article/10.3390/ijms26168007/s1.
