# Supplementary material for: Clinical and Proteomic Associations of SARS-CoV-2 Infection and COVID-19 Vaccination in Multimorbid Patients: A Cross-Sectional Observational Study
Source: Int J Mol Sci. 2025 Aug 19;26(16):8007. doi: 10.3390/ijms26168007 (PMC12387044; doi:10.3390/ijms26168007)
Supplement: Supplementary file 1 [file ijms-26-08007-s001.zip › ijms-3784522-supplementary.pdf]

Supplementary Materials

Clinical and Proteomic Associations of SARS-CoV-2 Infection and COVID-19 Vaccination in Multimorbid Patients: A Cross-Sectional Observational Study

Anett Hudák[1], Aladár Pettko-Szandtner[2], Annamária Letoha[3], and Tamás Letoha[1,*]

Supplementary Table S1. Summary of demographic and clinical characterization subgroups.

Figure Panel	Variable	Group	Mean	SD	SEM	95% CI Lower	95% CI Upper	Notes
1A	Age (years)	Men 18–35	27.41	4.214	1.022	25.25	29.58	Age × sex subgroup
		Women 18–35	26.46	4.493	0.849	24.72	28.21	
		Men 36–50	44.75	4.008	1.002	42.61	46.89	
		Women 36–50	43.97	4.547	0.844	42.24	45.70	
		Men 51–70	61.52	5.824	0.793	59.93	63.11	
		Women 51–70	62.54	5.790	0.819	60.89	64.19	
		Men >70	79.57	5.357	0.631	78.31	80.83	
		Women >70	81.93	7.772	0.777	80.39	83.47	
1B	Age (years)	COV+	65.19	19.74	1.109	63.01	67.37	$p = 0.0353$ vs. COV− patients
		COV−	58.78	20.05	2.864	53.02	64.53	
1C	Age (years)	COV+ vac−	66.93	20.31	2.084	62.79	71.06	Stratified by infection & vaccination status
		COV− vac−	58.44	23.92	7.972	40.06	76.83	
		COV+ vac+	64.66	19.40	1.302	62.10	67.23	
		COV− vac+	58.85	19.42	3.071	52.64	65.06	
1D	BMI (kg/m^2)	COV+ vac−	25.98	5.928	0.838	24.29	27.66	Variable differs from age in panels 1A–1C
		COV− vac−	22.42	3.912	1.749	17.56	27.27	
		COV+ vac+	27.30	5.610	0.423	26.47	28.14	
		COV− vac+	24.67	5.474	1.825	20.46	28.87	

Data are presented as mean ± standard deviation (SD), standard error of the mean (SEM), and 95% confidence intervals (CI). Subgroup definitions correspond to those shown in the indicated figure panels: Figure 1A – age × sex subgroups; Figure 1B – comparison of COVID-19–positive (COV+) vs. COVID-19–negative (COV–) patients; Figure 1C – infection/ vaccination strata (COV±, vac±); Figure 1D – BMI in infection/vaccination strata. p-values refer to between-group comparisons; significant differences are indicated where $p < 0.05$. BMI: body mass index; COV+: SARS-CoV-2–positive; COV–: SARS-CoV-2–negative; vac+: vaccinated; vac–: unvaccinated.

 https://doi.org/10.3390/ijms26168007

Supplementary Table S2. Details of the CALL score components and point system.

COVID-19 CALL score calculation		
Age factor	>60	3
	<60	1
Lymphocyte factor	≤1.0 × 10^9/L	3
	≥1.0 × 10^9/L	1
LDH factor	>500 U/L	3
	≥250 U/L	2
	<250 U/L	1
Comorbidity	yes	4
	no	1

Supplementary Table S3. Comorbidity associations with infection and vaccination

SARS-COV-2 infection vs. Comorbidity

Comorbidity	p-value	Odds Ratio	Interpretation
**CVD (Cardiovascular Disease)**	0.005	2.63	**Significant association**
**PMD (Psychiatric Disorders)**	0.3455	0.56	**Not significant**
**DM (Diabetes Mellitus)**	0.1629	2.14	**Not significant**; possible association
**CLD (Chronic Lung Disease)**	0.3374	0.64	**Not significant**
**ND (Neurological Disorders)**	0.2249	3.93	**Not significant**; high OR suggests potential trend
**HT (Hypertension)**	0.127	1.63	**Not significant**

Fisher's exact test results showing associations between SARS-COV-2 infection and comorbidities.

 https://doi.org/10.3390/ijms26168007

Supplementary Table S4. Laboratory parameters of deceased patients

Parameter	COV+ vac+ (mean ± SEM)	COV+ vac− (mean ± SEM)
**Age (years)**	82.2 ± 3.7	96 ± 7.1
**BMI**	28.23 ± 1.44	19.13 ± 0.4
**WBC (G/L)**	8.42 ± 0.93	13.63 ± 10.2
**Lymphocytes (G/L)**	0.74 ± 0.16	0.49 ± 0.08
**Lymphocyte (%)**	10 ± 3.5	9.15 ± 7.45
**Monocytes (G/L)**	0.64 ± 0.11	0.35 ± 0.05
**Monocyte (%)**	7.78 ± 1.3	3.35 ± 1.05
**Eosinophil (%)**	0.78 ± 0.45	0 ± 0
**Basophil (%)**	0.42 ± 0.09	0.05 ± 0.05
**Neutrophil (%)**	81 ± 4.9	87.45 ± 8.45
**RBC (T/L)**	4.01 ± 0.5	4.64 ± 0.59
**Hemoglobin (g/L)**	112 ± 8.2	123.5 ± 9.5
**Hematocrit (L/L)**	0.346 ± 0.03	0.385 ± 0.045
**Platelets (G/L)**	210 ± 50	481.5 ± 62.5
**MPV**	10.98 ± 0.5	9.9 ± 0.7
**MCHC (g/L)**	326 ± 7.5	320 ± 11
**Glucose (mmol/L)**	9.24 ± 2.09	8 ± 0.45
**Albumin (g/L)**	34.6 ± 4.1	6.85 ± 0.65
**LDH (U/L)**	287 ± 50	492.5 ± 20.5
**GOT (U/L)**	26 ± 5	66 ± 32
**GPT (U/L)**	12.6 ± 1.3	38 ± 8
**ALP (U/L)**	67.2 ± 7.9	136 ± 51
**GGT (U/L)**	17.8 ± 2.4	73.5 ± 44.5
**Sodium (mmol/L)**	135.6 ± 1.5	144 ± 11
**Potassium (mmol/L)**	4.98 ± 0.39	4.15 ± 1.85
**Creatinine (μmol/L)**	203.6 ± 56.9	233 ± 51
**eGFR (mL/min)**	42 ± 13.3	11 ± 4
**Urea (mmol/L)**	14.46 ± 1.33	18 ± 4
**CRP (mg/L)**	69.9 ± 22.8	199.5 ± 162.5
**IL-6 (pg/mL)**	166.1 ± 78.5	1106.5 ± 1065.5
**PCT (ng/mL)**	3.5 ± 3.13	27.75 ± 11.1
**Ferritin (ng/mL)**	324 ± 161	1109.7 ± 1045.75
**D-dimer (mg/L)**	2.66 ± 1.34	3.8 ± 1.15
**proBNP (ng/L)**	16065 ± 5544	9227.5 ± 481.5
**Troponin T (ng/L)**	189.6 ± 134.1	206.5 ± 120.5
**CK (U/L)**	236.8 ± 143.9	320 ± 185
**SpO_2 (%)**	92.9 ± 1.5	98.75 ± 1.25

Values are expressed as mean ± standard error of the mean (SEM). Five patients in the COV+ vac+ group and two patients in the COV+ vac− group died.

 https://doi.org/10.3390/ijms26168007

Supplementary Table S5.

	COV+ systole	COV- systole		COV+ diastole	COV- diastole
Number of values	317	49	Number of values	317	49
Mean	132,8	133,1	Mean	79,6	82,04
Std. Deviation	22,4	18,94	Std. Deviation	13,57	11,38
Std. Error of Mean	1,258	2,705	Std. Error of Mean	0,7619	1,626
	**vac+ systole**	**vac- systole**		**vac+ diastole**	**vac- diastole**
Number of values	262	104	Number of values	262	104
Mean	133,7	129,8	Mean	79,93	79,24
Std. Deviation	21,17	23,2	Std. Deviation	13,04	13,66
Std. Error of Mean	1,308	2,275	Std. Error of Mean	0,8055	1,34

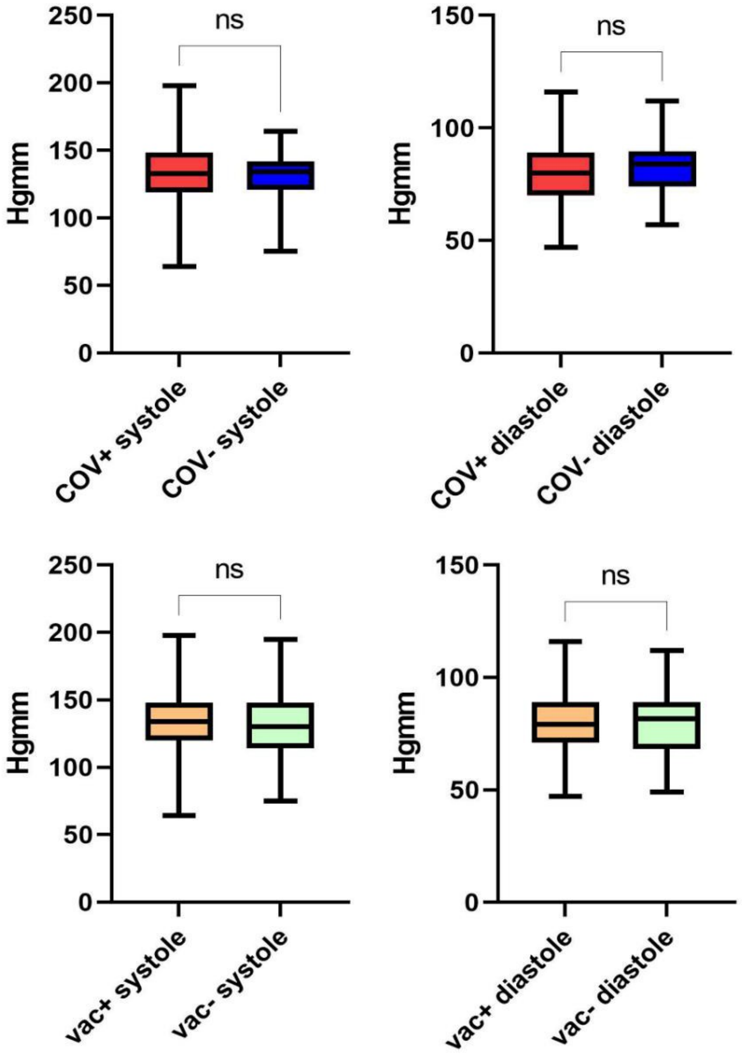

Supplementary Figure S1.

Supplementary Table S6. Pearson Correlation: vaccination and ferritin

Pearson r	
r	-0,1749
95% confidence interval	-0,2788 to -0,06685
R squared	0,03058
P value	
P (two-tailed)	0,0016
P value summary	**
Significant? (alpha = 0.05)	Yes
Number of XY Pairs	322

Pearson correlation analysis of blood parameters showing a significant negative correlation between vaccination status and ferritin levels.

Supplementary Table S7. Measurement units of patient blood parameters

Measured units name	Units of measure
D-dimer value	ug/ml
Creatinine level	umol/l
Ferritin level	ng/ml
IL-6 level	pg/ml
CRP level	mg/l
Troponin-T level	ng/ml
Lymphocyte count	%
Creatine-kinase	U/l
level proBNP level	pg/ml
spO_2 level	%
CALL-score level	points
Albumin level	g/l
LDH level	U/l
Carbamide level	mmol/l

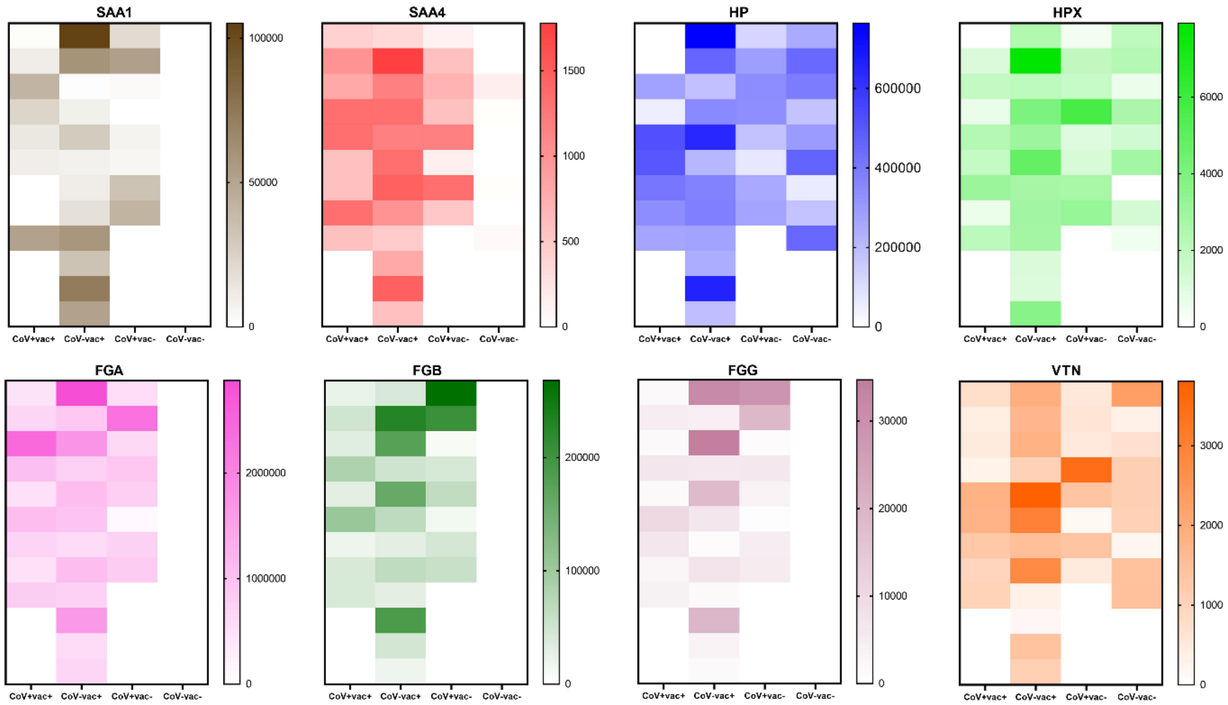

Supplementary Figure S2. MS-based heatmap of acute-phase proteins differentially expressed among COV+ vac+, COV− vac+, COV+ vac− and COV− vac+ patients. The color bar on the right side of the heatmap represents scaled expression values derived from MS analysis, with color gradients indicating relative protein abundance across samples. The scale increases from bottom to top, with darker colors representing higher expression levels.

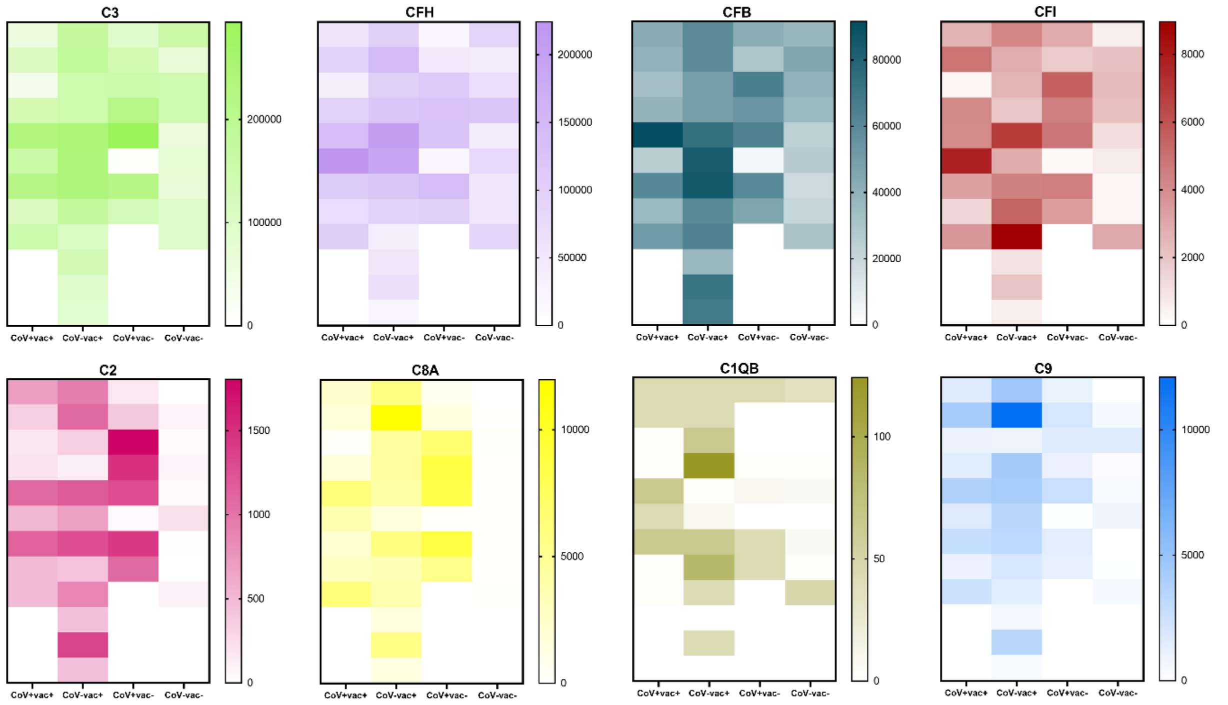

Supplementary Figure S3. Heatmap of complement system and immune response proteins identified by MS, showing differential expression across COV+ vac+, COV− vac+, COV+ vac− and COV− vac+ patientgroups. The color bar on the right represents scaled expression values, increasing from bottom to top, with darker colors indicating higher protein abundance.

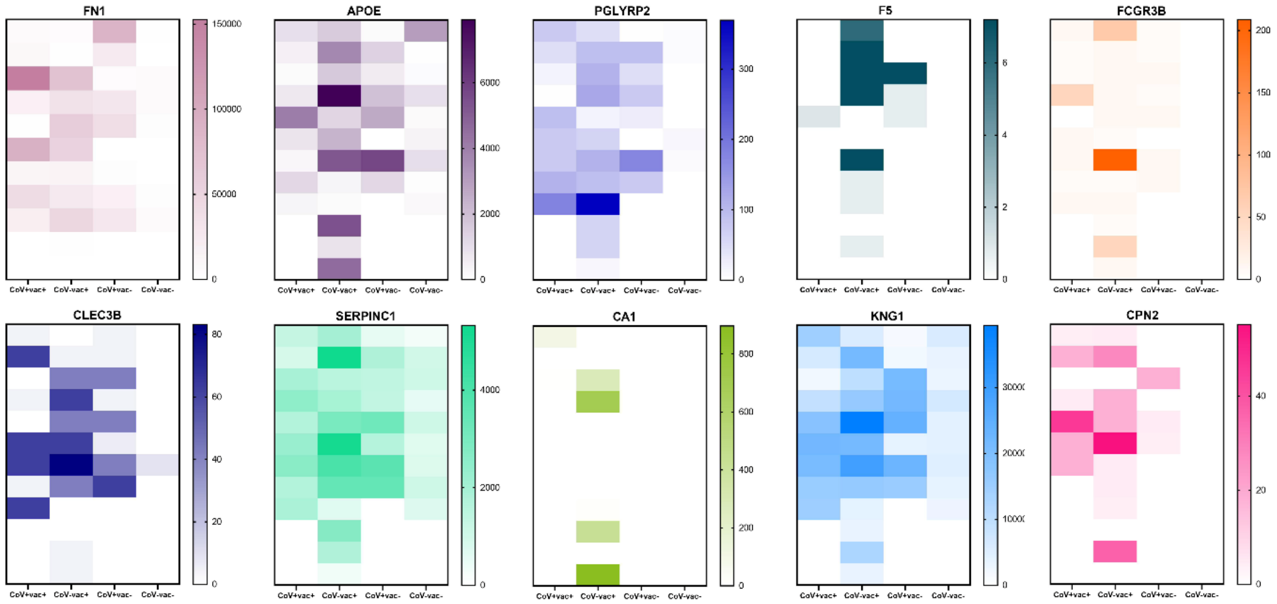

Supplementary Figure S4. MS-based heatmap of proteins related to coagulation, immunity, and extracellular interactions, showing differential expression across patient groups. The color bar on the right indicates scaled protein expression values derived from mass spectrometry analysis, increasing from bottom to top, with darker colors representing higher relative abundance.

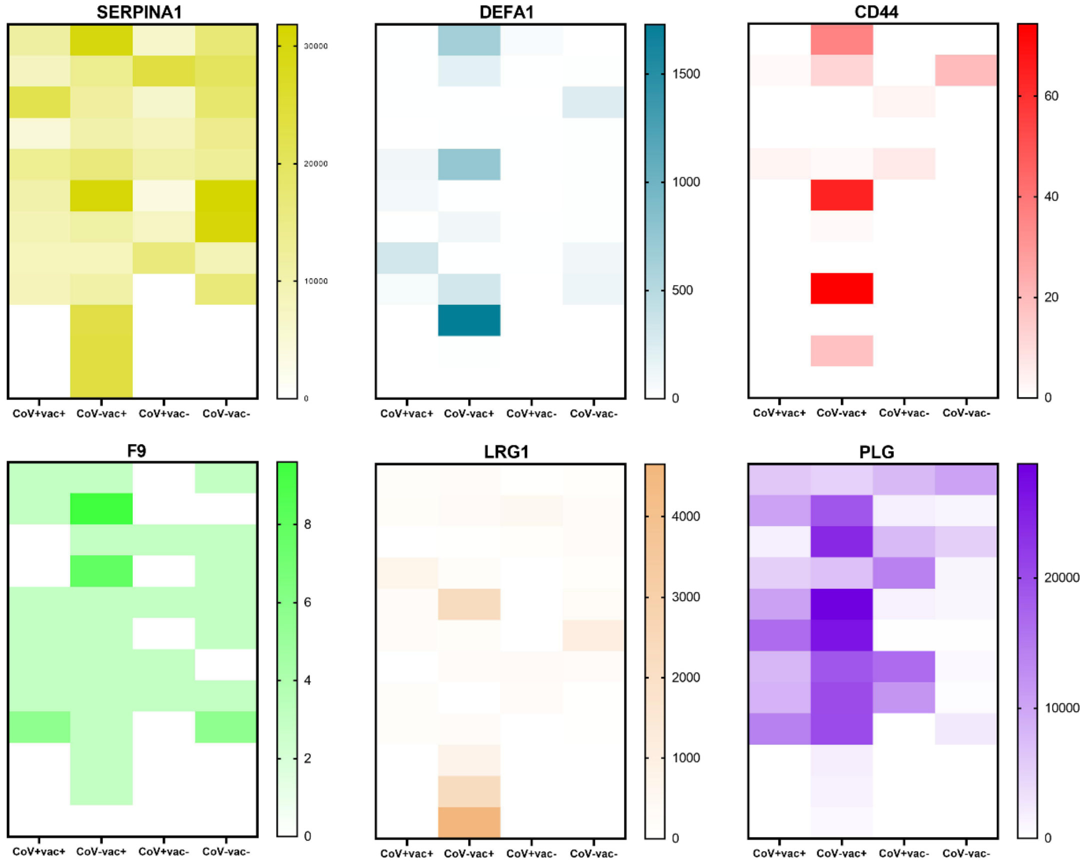

Supplementary Figure S5. Heatmap of proteins identified by MS analysis showing significant differences between COV+ vac+, COV− vac+, COV+ vac− and COV− vac+ individuals. The proteins shown are involved in various biological processes. The color bar on the right indicates scaled protein expression values derived from mass spectrometry analysis, increasing from bottom to top. Darker colors represent higher relative protein abundance.
